# Quantitative measurement of pseudoexfoliation in the anterior segment of the eye performed in visible light

**DOI:** 10.1186/1475-925X-12-74

**Published:** 2013-07-24

**Authors:** Robert Koprowski, Zygmunt Wróbel, Anna Nowinska, Edward Wylegala

**Affiliations:** 1 Faculty of Computer Science and Materials Science, Institute of Computer Science, Department of Biomedical Computer Systems, University of Silesia, ul. Będzińska 39, 41-200 Sosnowiec, Poland; 2 Department of Ophthalmology, District Railway Hospital, Katowice, Poland

**Keywords:** Automatic, Eye, Image processing, PEX, Pseudoexfoliation

## Abstract

**Introduction:**

Pseudoexfoliation syndrome (PEX) is a systemic disease involving the accumulation of pathological material deposits in the tissues of the anterior segment of the eye. The problem of modern ophthalmology is a quantitative assessment of the severity of PEX in the diagnosis and evaluation of the treatment progress in patients.

**Material and method:**

For the purposes of this study, 52 images of the anterior segment of the eye with the resolution of *M* × *N* = 1280 × 960 pixels were obtained in jpg format using the slit lamp CSO 450-SL. The patients were aged 50–80 and were recruited from Poland. All patients who participated in the study provided written informed consent after explanation of the nature and possible consequences of the study. The image analysis method proposed by the authors contains the calculation of the direction field, setting a straight perpendicular line passing through each pixel of the edge of the pupil, the calculation of the intersection of straight lines in order to determine the central point of the pupil position, the detection of the contour of PEX and the outer border of the iris with the use of the polar coordinate system. All analyzed parameters were set automatically with one exception parameter chosen manually depending on the slit lamp type.

**Results:**

A fully automatic measurement of PEX was carried out with the proposed method. Quantitative results enable to perform reproducible tests independently of the research centre. Owing to the image analysis method proposed by the authors, it is possible to obtain results in no more than 1 second on the Intel Core 2 Quad CPU 2.50 GHz with a measurement error below 3%. Other known methods of image analysis and processing that are compared in this paper give results with a greater error (4-35%) which depends on the degree of magnification (×6, ×16, ×20) and are not fully automatic.

**Conclusions:**

The methods of image analysis and processing enable a quantitative, repeatable and automatic measurement of the severity and progress of PEX syndrome. They support medical diagnosis and automatic archiving of results.

## Introduction

**Figure 1:**
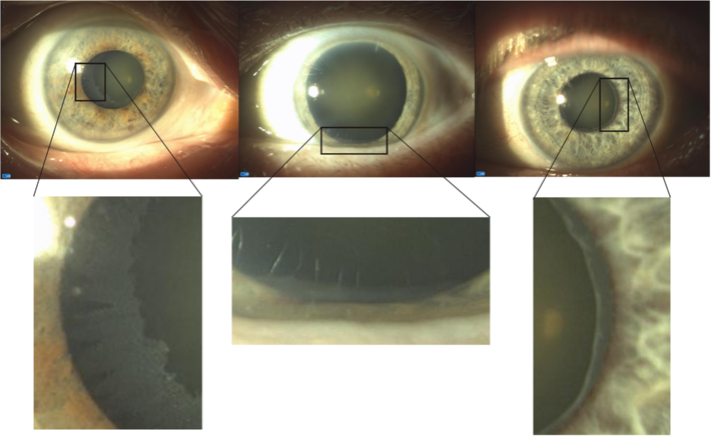
**Examples of images of the eye with pseudoexfoliation syndrome.** Pathological material deposits in tissues of the anterior segment of the eye are visible in all cases. The measurement of the severity of pseudoexfoliation should be performed regardless of the degree of mydriasis.

Pseudoexfoliation syndrome (PEX) is related to aging of the body. It is more common in people over 50 years of age, and in about 20% of the population above 60 years of age in the countries of northern Europe. PEX syndrome is a systemic disease and is recognized by an ophthalmologist during routine testing. Mydriasis and image analysis with a slit lamp enable to detect the nature and severity of PEX. This disease involves dysregulation of elastin synthesis and the formation of irregular elastic fibre aggregates, with a concomitant significant reduction of collagen fibres. Elastic changes are observed in the arterial and venous vessels and also around the sieve plates in the eye. Examples of images of the eye with PEX syndrome are shown in Figure 1.

The works on image analysis and processing of PEX images in ophthalmology are related to eye morphometric measurements performed in visible light. Morphometric analysis is presented in several works devoted to comparative analyses and in those associated with a selected region of the world. Quantitative parameters are presented in detail in the work of Seitz B. et al. from 1995 [1] on the analysis of images of the eyes with PEX performed for 85 images. However, the analysis in this work was not fully automatic - manual adjustment of the results with a mouse was provided. Patients with cataracts were analysed for the presence of PEX in the work of Upender K. Wali et al. in 2008 [2]. 69 preoperative cataract patients (43 males, 26 females) were analysed there. In the work of Bialasiewicz A.A. et al. from 2005 [3], 204 glaucoma eyes and 135 open-angle glaucomas were analysed. The incidence of PEX glaucomas relative to all glaucomas was 50.9% and to open-angle glaucomas 77%. This is one of many works concerning the analysis of PEX incidence in patients with glaucoma. Other known works refer to the quantitative analysis of patients from different areas of the world, for example, from Ethiopia [4], Greece [5], Iceland [6] or others [7] (the largest number of cases studied - 4430). Microscopy analysis presented in the work of Schlotzer-Schrehardt U.M. et al. [8] showed that eighty-five percent of the 85 PEX-eyes had endothelial cells polymegalism, 77% pleomorphism; 68% had white deposits and 42% had corneal guttae. White deposits and guttae were significantly more frequent and more intensive in PEX-eyes than in control eyes. PEX-eyes with and those without glaucoma studied in [8] showed no significant differences concerning the four qualitative parameters.

Due to the nature and shape of PEX syndrome [9-17], the methods of image analysis and processing which operate fully automatically offer different kinds of solutions. These include the active contour method [18], morphological methods or even simple binarization methods. It is also possible to use and profile well-known automatic methods of image analysis and processing from other branches of medicine [19-21]. These are for example: the methods of analysis of the eye tomographic images [22], the image analysis methods using the Hough transform [23,24], the methods of iridocorneal angle analysis in the images of the anterior segment of the eye [25] or other profiled methods of image analysis and processing [26-29]. The methods profiled for the analysis of non-parametric objects, that is PEX syndrome, are manual or semi-automatic [30]. An operator manually indicates the central point of the pupil position and then the algorithm draws the contour to the outline of PEX. In some cases, the operator also manually adjusts and corrects the algorithm errors. It follows that due to the quantitative reproducible assessment of the treatment progress, it is necessary to introduce fully automatic measurements. It is possible by profiling the algorithm strictly to the analysis of PEX syndrome. This type of algorithm is presented below.

## Material

For the purposes of this study, 52 images of the anterior segment of the eye with the resolution of *M* × *N* = 1280 × 960 pixels were obtained in jpg format using the slit lamp CSO 450-SL. The patients were aged 50–80 and they were all from Poland. All patients who participated in the study provided written informed consent after explanation of the nature and possible consequences of the study. In order to verify the sensitivity of the discussed method to the parameter changes, the images of the anterior segment of the eye were taken for 6-fold, 16-fold and 20-fold magnifications.

## Method

**Figure 2:**
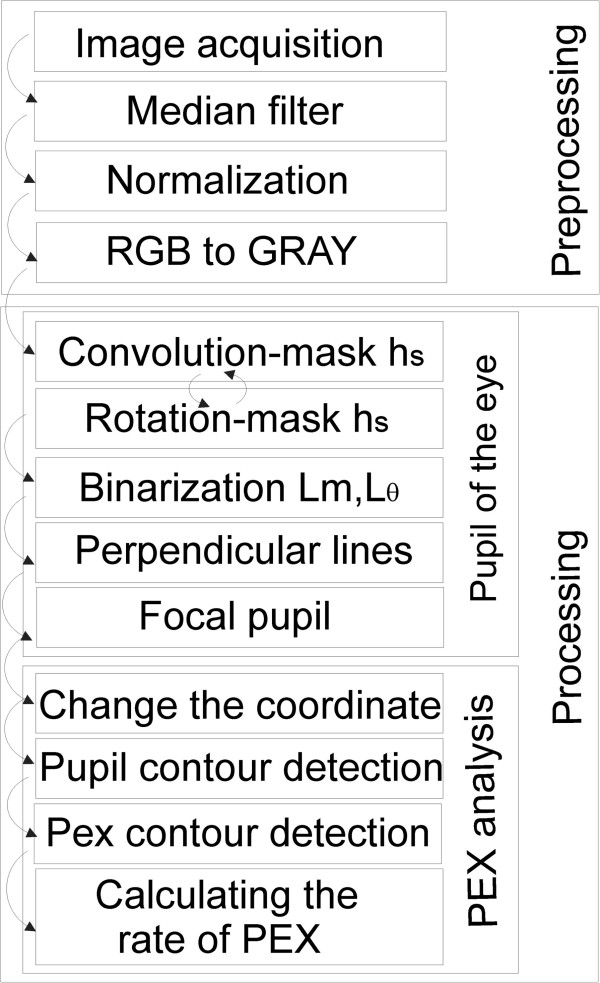
**Block diagram of the proposed algorithm for measuring the severity of PEX.** The algorithm consists of two main blocks – pre-processing and processing. The main image processing is in turn associated with two stages – determination of the pupil centre and final calculation of the severity of PEX syndrome.

**Figure 3:**
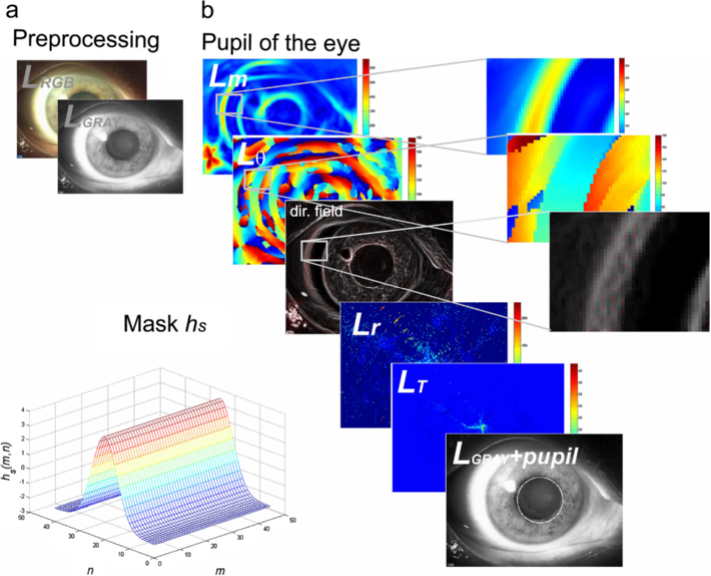
**Example of images obtained during first and second part of the proposed method of image analysis and processing.** The presented image processing parts were divided into two groups: image pre-processing **a)** determination of the position of the pupil centre **b)**. In some cases, magnifications of image fragments are shown.

**Figure 4:**
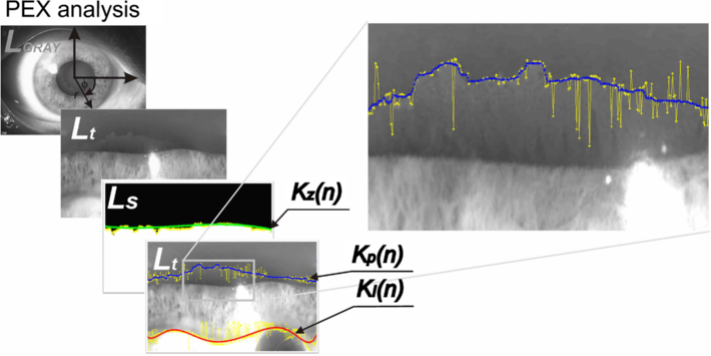
**Example of images obtained during a third part of the proposed method of image analysis and processing.** Previous two parts were shown on the Figure 3. The third part is the final part of the calculation of the severity of PEX syndrome. Initially the borders *K*_*z*_(*n*), *K*_*I*_(n) are estimated and the contour of PEX (*K*_*P*_(n)) is determined. Based on the obtained data the severity of PEX syndrome is calculated. In some cases, magnifications of image fragments are shown.

The method of image analysis and processing was divided into three parts – Figure 2. The first part concerns image pre-processing. The second one describes the method of determining the central point of the pupil position. The third part describes the method for translating an image to a different coordinate system and the final measurement of PEX. The results obtained in the subsequent stages, which are described below, are shown in Figures 3 and 4.

### Pre-processing

Image pre-processing involves the removal of noise and normalization of brightness levels. The input image *L*_*RGB*_(*m,n,k*) (where *m* – rows, *n* – columns, *k* – components R, G or B) derived from the slit lamp CSO 450-SL is subjected to median filtering using a mask *h*_*e*_ sized *M*_*he*_ × *N*_*he*_ = 3 × 3 pixels [19]. Then, there follows normalization of the individual components R,G,B to the range 0–1. The image *L*_*W*_(*m,n,k*) obtained in this way is converted to gray levels *L*_*GRAY*_(*m,n*) = 0.2989*L_*W*_(*m,n*,1) + 0.5870*L_*W*_(*m,n*,2) + 0.1140*L_*W*_(*m,n*,3) [20,21]. The resulting image *L*_*GRAY*_(*m,n*) is subjected to further image analysis (Figure 3a).

### Image processing

The main image processing algorithm consists of two stages - Figures 3b and 4. These concern determination of the position of the pupil centre and the contour of PEX syndrome. In the initial stage of processing, the *L*_*GRAY*_(*m,n*) image convolution with the *h*_*s*_(*m,n*) mask is calculated. The size of the mask *h*_*s*_ is chosen once for a given type of the slit lamp. In the analysed case (slit lamps CSO 450-SL), it is *M*_*hs*_ × *N*_*hs*_ = 45 × 45. The mask *h*_*s*_ is shown in Figure 3b. The mask *h*_*s*_ is rotated sequentially in the angular range *θ*∈[0, 180) by increments of 1°, 5°, or 10°. Small values of increments increase the computation time and accuracy, and vice versa, larger values reduce the accuracy and computation time. In each step of the rotation of *h*_*s*_ mask, the weave with the *L*_*GRAY*_(*m,n*) image is calculated and two matrices, namely *L*_*m*_(*m,n*) and *L*_*θ*_(*m,n*), are formed. The first marix *L*_*m*_(*m,n*) contains the maximum pixel values obtained in each weave. The matrix *L*_*θ*_(*m,n*), on the other hand, contains the angular values showing for what angle *θ* of rotation of the *h*_*s*_ mask, it occured. On the basis of the matrices *L*_*m*_(*m,n*) and *L*_*θ*_(*m,n*), it is possible to calculate the direction field shown in Figure 3b. Then, when setting a straight perpendicular line passing through each pixel with a slope of *θ + 90*°, the intersection of straight lines is calculated. As a consequence, two matrices *L*_*T*_(*m,n*) and *L*_*r*_(*m,n*) are formed. The first one (*L*_*T*_(*m,n*)) contains information about the intersection of straight lines. The value of a given pixel in the matrix *L*_*T*_(*m,n*) represents the number of intersections of the straight lines at this point (Figure 3b). The other matrix (*L*_*r*_(*m,n*)) contains information about the radius calculated from a given pixel to the intersection (Figure 4). The position of the pupil centre is therefore at the location (m*,n*) of the maximum value in the matrix *L*_*T*_(m*,n*) = max(*L*_*T*_(*m,n*)). The pupil diameter is a value read from the matrix *L*_*r*_(m*,n*). This information is necessary to perform subsequent image analysis operations in the last step (Figure 4) of determining the numerical values of PEX syndrome.

In order to calculate the severity of PEX syndrome, it is necessary to detect the contour of PEX and the outer border of the iris. With the value of the pupil radius *r*_*z*_ and the position of the pupil centre (m*,n*), conversion to polar coordinates is performed, which results in the image *L*_*ϕ*_(*r,θ*) (Figure 4). The change of the coordinate system facilitates detection of contours and their proper localization. In order to determine the contour of the pupil *K*_*z*_(*n*), the image *L*_*t*_(*m,n*) was subjected to binarization, followed by the method of filling the holes. The resulting image *Ls*(*m,n*) enables to detect the pupil edge position. Its position is further approximated by a polynomial (yellow and green in Figure 4). Then, the contour of PEX syndrome *K*_*P*_(*n*) and the contour of the outer boundary of the iris *K*_*I*_(*n*) are determined using the method of the modified active contour [22]. The results obtained (*K*_*z*_(*n*), *K*_*P*_(*n*), *K*_*I*_(*n*)) are shown in the images *L*_*s*_(*m,n*) and *L*_*t*_(*m,n*) in Figure 4, marked in green, blue and red respectively. The value of the severity of PEX syndrome was calculated as:

(1) $$W_{\mathrm{PEX}}=\sum_{n}\frac{K_{I}\left(n\right)-K_{P}\left(n\right)}{K_{I}\left(n\right)}*100\%$$

The defined value *W*_*PEX*_ enables to obtain reliable results only in the case of a fully visible contour *K*_*P*_(*n*). It is therefore necessary to check the following condition in each case:

(2) $$W_{1/0}=\left\{\begin{array}{lll} 0 & \mathrm{if} & \min_{n}\left(K_{Z}\left(n\right)-K_{P}\left(n\right)\right)>0\\ 1 & \mathrm{if} & \min_{n}\left(K_{Z}\left(n\right)-K_{P}\left(n\right)\right)\leq 0 \end{array}\right)$$

When the pupil contour coincides with the set contour of PEX (*W*_*1/0*_ = 0), the measurement is considered to be unreliable.

### The analysis of the obtained results

**Figure 5:**
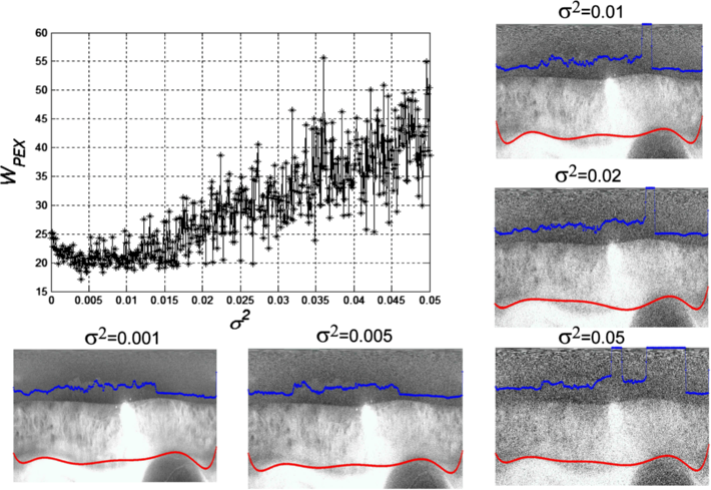
**A plot of a function that displays the change of the *W***_***PEX***_**value dependent on the increase of the noise level (variance *σ***^***2***^**) on the input image *L***_***RGB***_**.** Additionally representative examples of the results obtained for the same patient for the selected variance (0.001, 0.005, 0.01, 0.02 and 0.05) were shown. Contours *K*_*P*_(*n*) and *K*_*I*_(n) obtained for the selected level of noise were marked blue and red respectively (analogous to Figure 4).

The proposed method and algorithm was used for analysis of 52 images. Representative examples of obtained results and image processing parts were presented on Figures 3 and 4. The algorithm is fully automatic. The only manually chosen parameter is the size of the mask *h*_*s*_ (for a given type of the slit lamp). However, the quality of the obtained image of the PEX contour has the greatest impact on the results. In many cases the image analysis had to be conducted despite the high level of noise. The image noise was usually caused by disturbances of the measurement path (optical), small inclusions, dust, etc. The observed image disturbances were characterized as Gaussian noise. Therefore, a verification of the results obtained according to the artificial noise added to the image *L*_*RGB*_ was conducted. The artificial noise was the Gaussian noise with zero mean value and the variance *σ*^*2*^ ranged from 0 to 0.05 at 0.001. The Figure 5 contains a plot of a function that displays the change of the *W*_*PEX*_ value dependent on the increase of the noise level (variance *σ*^*2*^) on the input image *L*_*RGB*_. Additionally the Figure 5 contains representative examples of the results obtained for the same patient for the selected variance (0.001, 0.005, 0.01, 0.02 and 0.05). The obtained results were significantly disturbed by the increasing of the noise level. An almost double increase of the *W*_*PEX*_ value was noticed when the variance value exceeded 0.035 (Figure 5). Further increasing the level of noise added to the image *L*_*RGB*_ further aggravated this result (increase of the *W*_*PEX*_ value). In this presented case the reason for measurement errors was incorrectly determined contour *K*_*P*_(*n*) – Figure 5 marked blue. This is the result of the insufficient visibility of the PEX contour, which made an accurate measurement impossible. It should be noted that adding noise to the image do not significantly change the accuracy of the *K*_*I*_(n) contour determination (marked red). The reason is a sufficient contrast between the iris and the sclera and thus little influence of noise on the obtained results.

The comparison among other known methods with the proposed methods is presented in the next part.

## Results and their comparison with the results obtained with known methods

The discussed measurement method of PEX syndrome was compared with two other methods. The results were referred to the contours marked by a specialist physician. As a result, a comparison was made of the value *W*_*PEX*_ determined automatically by three methods with the value *W*_*PEXE*_ determined by a specialist physician. Denoting the subsequent values *W*_*PEX*_ as *W*_*PEXM*_, where *M* - method number, an error for each method was calculated:

(3) $$\delta_{\mathrm{PEXM}}=\frac{\left|W_{\mathrm{PEXM}}-W_{\mathrm{PEXE}}\right|}{W_{\mathrm{PEXE}}}*100\%$$

The comparison of the quality of the results was performed based on the following methods:

**Figure 6:**
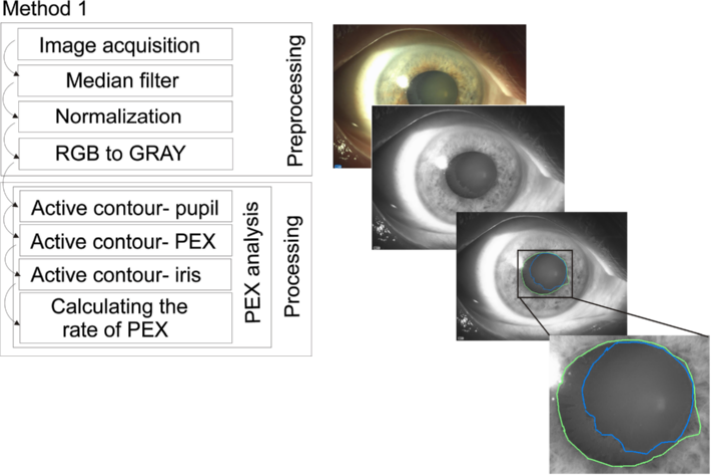
**Block diagram of the first described image analysis and processing method compared to the proposed method.** This method (Method 1) is based on the active contour and discussed in a published paper [18]. The main disadvantages of these method are not fully automatic measurements and lack of measurement reproducibility.

Method 1 (*W*_*PEX1*_) – image pre-processing is the same as for the described case (median filtering, normalization to the full range of gray levels). Proper analysis concerning determination of the contours of PEX, the pupil and the iris is based on the active contour method proposed by Wu H. et al. in [18]. Nodes in this method are set manually by an operator. The number of set points is not strictly defined. Typically, a physician introduces 4–6 points in the pupil area. The block diagram of this method is shown in Figure 6.

**Figure 7:**
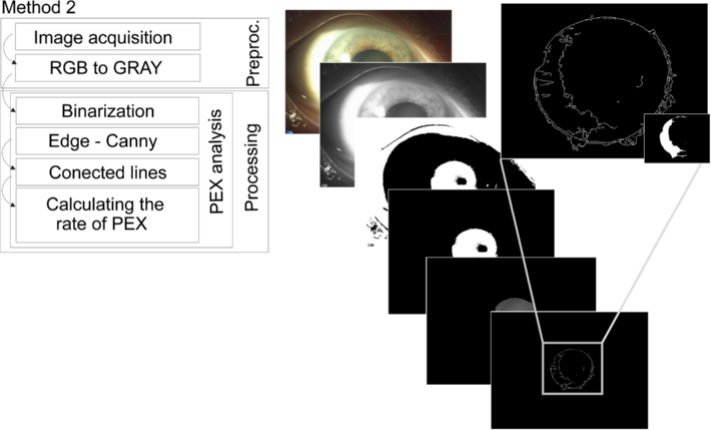
**Block diagram of the second described image analysis and processing method compared to the proposed method.** This method (Method 2) is based on the Canny contour and discussed in a published paper [31]. The main disadvantages of these method, analogously to method 1 are not fully automatic measurements and lack of measurement reproducibility.

Method 2 (*W*_*PEX2*_) – image pre-processing only involves conversion of an ego colour image into a grayscale image. Proper analysis is related to binarization which is necessary to pre-determine the position of the pupil. The resulting binary image is used as a mask on which the contour is determined using Canny method [31]. The resulting contour fragments are not continuous. These are further connected to each other by straight lines. The block diagram of this method is shown in Figure 7.

Method 3 (*W*_*PEX3*_) – described in this paper.

**Figure 8:**
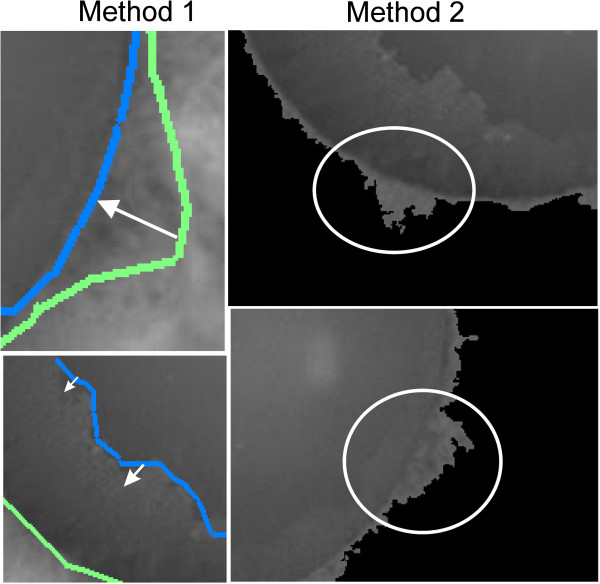
**Wrong results obtained for the compared methods 1 and 2.** Method 1 (first column) does not work well when the parameters of the active contour are chosen incorrectly or the points are wrongly indicated by an operator. Method 2 (second column) cannot cope in the event of improper illumination of the eye. In these cases, it incorrectly identifies the pupil contour during binarization. The interesting fragments are marked with white arrows and areas.

The three methods were implemented and the value of the error *δ*_*PEXM*_ was measured for each method for the analysed 52 images. Calculations were carried out for different magnifications: ×6, ×16 and ×20. The results are shown in Tables 1 and 2. The analysis of the obtained data shows that the proposed method 3 introduces the smallest measurement error for the 10-fold magnification, that is *δ*_*PEX3*_ = 3%. The largest error is introduced by the method 2, namely *δ*_*PEX3*_ = 25%. Such a large error value is due to the adopted methodology, that is the sequence of operations in each block (Figure 7). The procedure for the image analysis in the method 2 leads to the formation of numerous artefacts and errors – examples are shown in Figure 8. Similarly, the method 1 leads to errors related to matching of the active contour to the edges of the pupil and iris (Figure 8). The correct position of these edges is often skewed by, for example, reflections of the light source. The set magnification (×6, ×16 and × 20) affects, to a large extent, the accuracy obtained by the methods 2 and 3. This is due to the size of the pupil in the scene. For small magnifications, the pupil is relatively small in the scene. For large magnifications, the pupil is not fully visible, or due to the focal length, the image is blurred. The result is that for 16-fold magnification (Table 2), the error obtained is the smallest for all the considered methods.

**Table 1:** **Summary of the results obtained for the compared methods for a sample image (*W***_***PEXE***_ **= 24%)**

**Magnification**	***W***_***PEX1***_	***W***_***PEX2***_	***W***_***PEX3***_
**×6**	25%	30%	25%
**×16**	25%	28%	25%
**×20**	25%	34%	26%

**Table 2:** Summary of average values of errors obtained for all images for all the compared methods

**Magnification**	***δ***_***PEX1***_	***δ***_***PEX2***_	***δ***_***PEX3***_
**×6**	5%	25%	4%
**×16**	5%	17%	3%
**×20**	6%	35%	7%

## Conclusions

This paper presents a quantitative, fully automatic method for the assessment of the severity of pseudoexfoliation syndrome. The advantages of the presented method are:

– reproducibility of measurements for large interindividual variability of patients,

– information for the physician concerning the quality of the measurement (*W*_*1/0*_ ratio),

– measurements with an error of less than 3% for 6-fold and 16-fold magnifications,

– correct algorithm operation in different lighting conditions,

– setting all the parameters automatically depending on the image resolution,

– lack of impact of physician’s individual settings of the slit lamp on the results obtained,

– lack of impact of the slit lamp type on the results obtained (owing to the full automation of measurements),

– single image analysis time in under 1 second on the computer Intel Core 2 Quad CPU Q9300 @ 2.GHz RAM 8GB.

The practical usefulness of this method has been confirmed by specialist physicians. It is used in many branches of ophthalmology supporting medical diagnostics or enabling clinical trials. It is now being implemented in the Department of Ophthalmology in District Railway Hospital in Katowice, Poland.

Further papers of the authors will focus on the analysis of the correlation between other ocular parameters such as intraocular pressure or visual field and the severity of the pseudoexfoliation syndrome.

## Abbreviations

PEX: Pseudoexfoliation.

## Competing interests

The authors declare that they have no competing interests.

## Authors’ contributions

RK and ZW suggested the algorithm for image analysis and processing, implemented it and analysed the images. AN, EW performed the acquisition of the 52 images and consulted the obtained results. All authors have read and approved the final manuscript.
